# The Left Ventricular Outflow Tract Velocity Time Integral as a Predictor of Fluid Responsiveness in Patients With Sepsis-Related Acute Circulatory Failure

**DOI:** 10.7759/cureus.77353

**Published:** 2025-01-12

**Authors:** Parvathy Sasidharan, Nidhi Kaeley, Pankaj Sharma, Gaurav Jain, Takshak Shankar, Sreejith Jayachandran, Balwant Kumar, Mallapu Ajay Kumar, Jewel Rani Jose, Devinder Kumar Lalotra

**Affiliations:** 1 Emergency Medicine, All India Institute of Medical Sciences, Rishikesh, Rishikesh, IND; 2 Radiology, All India Institute of Medical Sciences, Rishikesh, Rishikesh, IND; 3 Anesthesiology, All India Institute of Medical Sciences, Rishikesh, Rishikesh, IND

**Keywords:** fluid responsiveness, ivc collapsibility index, lvot vti, point-of-care ultrasound (pocus), septic shock (ss)

## Abstract

Background: Sepsis is a major contributor to global morbidity and mortality. Effective fluid resuscitation is essential for managing septic shock, but it must be carefully monitored to avoid fluid overload and related complications. Recent studies have demonstrated that both inadequate and excessive fluid resuscitation are linked to poor outcomes.

Methods: This observational study was conducted over 18 months, including spontaneously breathing patients aged 18 to 65 with sepsis-related acute circulatory failure. Patients were enrolled through convenience sampling. Baseline vital signs and point-of-care ultrasound (POCUS) parameters were recorded. A volume expansion test (VET) was performed, administering 500 ml of normal saline over 15 minutes, followed by reassessment of vital signs and POCUS parameters. Patients were classified as responders or non-responders. The study evaluated the left ventricular outflow tract velocity time integral (LVOT VTI) as a predictor of fluid responsiveness.

Results: The study enrolled 113 patients with a mean age of 48.69 years (SD: ±16.81). The most common age group was 61-70 years (24 patients; 21.2%), and there was a male predominance (73 patients; 64.6%). Forty-eight patients (42.5%) had no comorbidities, with hypertension being the most prevalent (17 patients; 15.0%). Pneumonia was the most common source of sepsis (50 patients; 44.2%), and 16 patients (14.2%) died. The percentage change in LVOT VTI following the VET demonstrated a sensitivity of 96.0% and specificity of 100%, with an area under the receiver operating characteristic (ROC) curve of 0.992. A percentage change of ≥15.19% indicated high fluid responsiveness, although a single VTI measurement alone was not a reliable predictor.

Conclusion: LVOT VTI measurements play a critical role in assessing fluid responsiveness in sepsis-related acute circulatory failure. While a single VTI measurement is unreliable, the percentage change in LVOT VTI after a VET offers excellent diagnostic performance. A cutoff of ≥15.19% post-expansion indicates a high likelihood of fluid responsiveness.

## Introduction

Sepsis is a critical concern for emergency physicians, as it is a potentially fatal condition that causes organ dysfunction due to an inappropriate immune response to infection [1]. Septic shock is defined as a subset of sepsis in which particularly profound circulatory, cellular, and metabolic abnormalities are associated with a greater risk of mortality than with sepsis alone and who are clinically identified by a vasopressor requirement to maintain a mean arterial pressure (MAP) of 65 mm Hg or greater and serum lactate level greater than 2 mmol/L (>18 mg/dL) in the absence of hypovolemia [2]. Mortality rates range from 15% to 56% in the case of septic shock [3]. Effective fluid resuscitation is essential, as both insufficient and excessive fluid administration can lead to adverse outcomes [4,5]. Traditional static measures often fail to accurately predict fluid responsiveness, emphasizing the need for more precise methods. Dynamic preload indicators can help identify patients who are likely to benefit from fluid therapy, thus reducing unnecessary fluid administration. Non-invasive techniques, such as point-of-care ultrasound (POCUS), are crucial for evaluating hemodynamic changes, and assessing the inferior vena cava (IVC) can provide valuable insights into volume status, although its reliability varies across different patient populations [6].

The left ventricular outflow tract velocity time integral (LVOT VTI) serves as a surrogate marker for stroke volume, calculated from the Doppler spectrum across the outflow tract valves. Stroke volume can be determined by multiplying VTI by the cross-sectional area of an anatomical site, with increases in VTI indicating higher cardiac output. Studies have demonstrated the predictive value of LVOT VTI in intensive care and postoperative settings [7,8]. For instance, Xie et al. [7] showed the strong predictive value of LVOT VTI variation in assessing fluid responsiveness in post-operative mechanically ventilated patients. With an impressive area under the receiver operating characteristic curve (AUROC) of 0.919, LVOT VTI variation was proved to be a reliable indicator for predicting fluid responsiveness at an optimal cutoff value of 12.51% for VTI variation; the sensitivity and specificity were 71.9% and 75.0%, respectively. The research about LVOT VTI in emergency departments remains limited, often focusing on mechanically ventilated patients. This study aims to evaluate the diagnostic performance of LVOT VTI in the emergency department, contributing to more effective fluid resuscitation and potentially improving outcomes for patients in the early stages of sepsis treatment. The primary objective of the study was to assess the role of LVOT VTI as a predictor of fluid responsiveness in patients with sepsis-related acute circulatory failure, and the secondary objective was to compare the baseline variables among the responders versus non-responders to resuscitation in patients presenting with sepsis-related acute circulatory failure.

## Materials and methods

The efficacy of LVOT VTI as a predictor of fluid responsiveness in patients with sepsis-related acute circulatory failure was evaluated in our observational study conducted in the Department of Emergency Medicine at All India Institute of Medical Sciences, Rishikesh, India. Over an 18-month period, 90 spontaneously breathing patients aged 18 to 65, who were diagnosed with sepsis according to SEPSIS-3 criteria [1], were recruited using convenience sampling. Inclusion criteria required a MAP of less than 65 mmHg, systolic blood pressure (SBP) below 90 millimeters of mercury (mmHg), tachycardia exceeding 100 beats per minute, or elevated serum venous lactate levels greater than 2 millimoles per liter [9]. Exclusion criteria included a body mass index (BMI) over 30 kilograms per meter square, the presence of cardiac arrhythmias, a history of myocardial infarction, valvular heart disease, cardiomyopathy, inadequate transthoracic imaging, obstructive shock, mechanical ventilation, refusal to consent, chronic kidney disease with creatinine clearance less than 30 milliliters per minute, patients on maintenance dialysis, and pregnancy.

Ethical approval and consent to participate

The study was approved by the Institutional Ethics Committee, All India Institute of Medical Sciences, Rishikesh (AIIMS/IEC/23/314). All patients enrolled in the study signed an informed written consent for the use and publication of their medical records for academic and research purposes.

Sample size calculation

The sample size for our study was calculated using MedCalc software version 20.123 (MedCalc Software Ltd., Belgium). With an alpha error of 5%, a power of 80%, and an expected ROC area under the curve (AUC) of 0.84 for the primary variable (LVOT VTI), we aimed to demonstrate statistical significance against a null hypothesis AUC of 0.5 (based on initial pilot observations). The calculated sample size was 22. Expecting a dropout rate of 10%, the calculated sample size was 24.

Methods

Demographic parameters and clinical histories were collected from all patients, who then underwent a clinical examination with documented findings. Baseline parameters, including blood pressure, heart rate (HR), mean arterial pressure (MAP), pulse pressure, and peripheral oxygen saturation (SpO₂), were recorded. Additional tests included arterial blood gas analysis, serum lactate levels, complete blood count (CBC), liver and kidney function tests, and procalcitonin levels, which were done as part of the sepsis workup. A standard 12-lead electrocardiogram (ECG) was performed, and POCUS was used to screen the lungs, abdomen, and heart.

**Figure 1:**
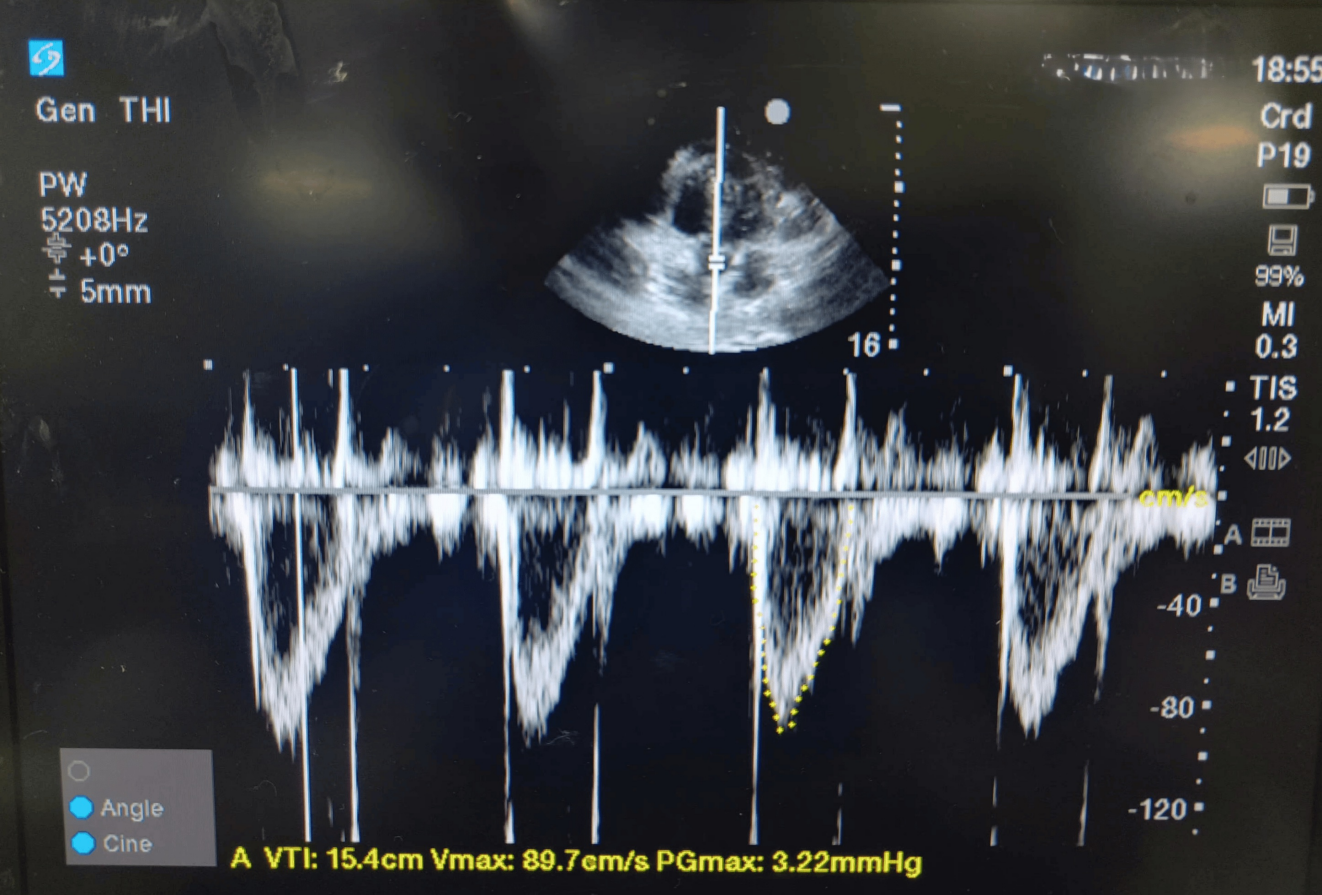
Measurement of LVOT VTI LVOT: left ventricular outflow tract; VTI: velocity time integral

The LVOT VTI was measured using the apical five-chamber view obtained from the apical four-chamber view. With the patient in the supine position, the transducer was positioned below the left breast, and the image was optimized to display all four chambers. The ultrasound beam was tilted anteriorly to visualize the LVOT, aortic valve, and proximal ascending aorta, with the pulse wave Doppler sample volume placed 5 mm proximal to the aortic valve. The spectral signal, characterized by a rapid upstroke and end-systolic click, was traced to calculate the VTI (Figure 1).

**Figure 2:**
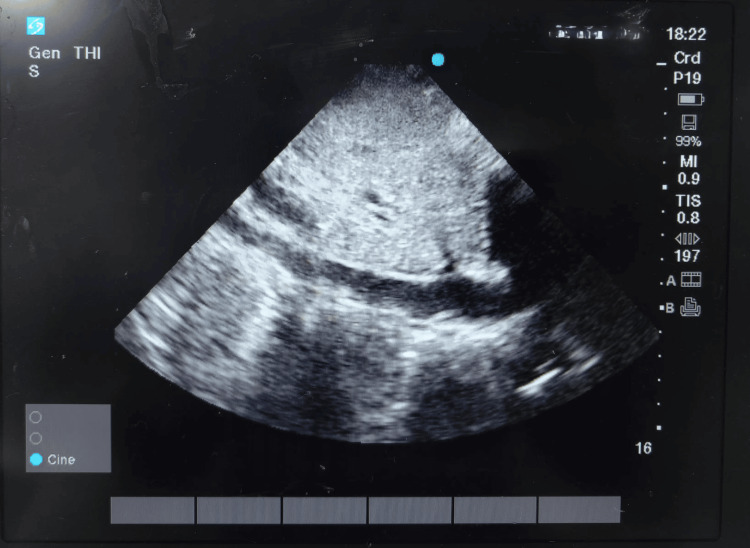
Inferior vena cava (IVC) in the subxiphoid view

The normal range for LVOT VTI is typically between 18 cm and 22 cm, and for HRs, it is between 55 bpm and 95 bpm [10]. All participants underwent a volume expansion test, receiving 500 mL of normal saline over 15 minutes. Patients were classified as responders or non-responders based on the IVC collapsibility index (IVCCI), with responders defined as having an IVCCI ≥ 40% [11]. To measure the IVCCI using the subcostal view, we began by visualizing the heart with a phased-array probe with the patient in the supine position. The probe was rotated vertically, with the orientation marker pointing cranially, and shifted 1 to 2 cm to the right of the patient's midline, ensuring the right atrium (RA) remained visible to obtain a view of the IVC along its long axis. The IVC was located to the right of the midline, and the aorta and the IVC were assessed 3 to 4 cm from the RA or 1 cm distal to the hepatic vein (Figure 2).

The maximum and minimum IVC diameters during respiration were measured. The collapsibility index (CI) was calculated as \[ \text{CI} = \frac{\text{IVC}_{\text{max}} - \text{IVC}_{\text{min}}}{\text{IVC}_{\text{max}}} \] While IVC CI <20% (with moderate to large IVC diameter) with spontaneous inspiration or mechanical ventilation suggests elevated mean RAP ≥15 mmHg, IVC maximum diameter <2.1 cm that collapses >50% with or without a sniff suggests normal mean RAP 0 mmHg to 5 mmHg [12]. After the test, vital and POCUS parameters were reassessed to evaluate LVOT VTI as a predictor of fluid responsiveness.

Statistical analysis

Data analysis was performed using IBM SPSS Statistics for Windows, Version 25 (Released 2017; IBM Corp., Armonk, New York, United States). Categorical variables were presented as numbers and percentages, while continuous variables were reported as mean (SD) or median (interquartile range (IQR)), depending on the data distribution assessed by the Shapiro-Wilk test. Categorical variables were analyzed using the chi-squared test or Fisher’s exact test, while continuous variables were compared using the Wilcoxon-Mann-Whitney U test or t-test. ROC curves were plotted for LVOT VTI values, and the area under the ROC curve (AUROC) was calculated. Statistical significance was set at p < 0.05.

## Results

The study enrolled 113 patients with a mean age of 48.69 years (±16.81). The largest age group was 61-70 years (21.2%), and 64.6% (n = 73) of participants were male. Among the cohort, 42.5% had no comorbidities, 15.0% had hypertension, and 12.4% had both diabetes and hypertension. The mean sequential organ failure assessment (SOFA) score was 10.81 (±1.52), the mean quick sequential organ failure assessment (qSOFA) score was 1.78 (±0.98), and the mean modified early warning score (MEWS) was 6.74 (±1.72) (Table 1).

**Table 1:** Demographic parameters of all patients in the study population SOFA: sequential organ failure assessment; qSOFA: quick sequential organ failure assessment; MEWS: modified early warning score; GCS: Glasgow coma scale; SD: standard deviation; IQR: interquartile range

Parameters	Mean ± SD/median + IQR/number (%)
Age (years)	48.69 ± 16.81
Age	
18-30 years	22 (19.5%)
31-40 years	19 (16.8%)
41-50 years	17 (15.0%)
51-60 years	20 (17.7%)
61-70 years	24 (21.2%)
71-80 years	9 (8.0%)
81-90 years	2 (1.8%)
Gender	
Male	73 (64.6%)
Female	40 (35.4%)
Comorbidities	
None	48 (42.5%)
Hypertension	17 (15.0%)
Diabetes mellitus, hypertension	14 (12.4%)
Diabetes mellitus	13 (11.5%)
Coronary artery disease	8 (7.1%)
Hypothyroidism	8 (7.1%)
Chronic liver disease	5 (4.4%)
SOFA	10.81 ± 1.52
qSOFA	1.78 ± 0.98
MEWS	6.74 ± 1.72
GCS	13.91 ± 1.04
Temperature (degree Celsius)	37.11 ± 1.14

The sources of sepsis included pneumonia (44.2%, n = 50), urinary tract infections (UTIs) (20.4%, n = 23), and neutropenic infections (10.6%, n = 12). Other sources included bloodstream infections (9.7%, n = 11), intra-abdominal infections (7.1%, n = 8), meningitis (4.4%, n = 5), and necrotizing soft tissue infections (3.5%, n = 4) (Table 2).

**Table 2:** Details of the source of sepsis among the study participants NSTI: necrotizing soft tissue infection; UTI: urinary tract infection

Source of sepsis	Frequency	Percentage
Pneumonia	50	44.2%
UTI	23	20.4%
Neutropenic infection	12	10.6%
Bloodstream infection	11	9.7%
Intra-abdominal infection	8	7.1%
Meningitis	5	4.4%
NSTI	4	3.5%

Outcomes revealed that 30 participants (26.5%) were discharged, 33 (29.2%) required intensive care unit (ICU) admission, 22 (19.5%) needed mechanical ventilation, and 12 (10.6%) required hemodialysis. Unfortunately, 16 participants (14.2%) did not survive (Table 3).

**Table 3:** Distribution of outcome among the study population ICU: intensive care unit

Outcome	Frequency	Percentage
Discharge	30	26.5%
ICU admission	33	29.2%
Need for mechanical ventilation	22	19.5%
Need for hemodialysis	12	10.6%
Dead	16	14.2%

Significant variables associated with mortality included qSOFA (W = 1158.500, p < 0.001), MEWS (W = 1482.000, p < 0.001), and GCS (W = 286.500, p < 0.001), all showing higher median scores in the mortality group. Survivors exhibited higher SBP (W = 390.500, p = 0.001) and diastolic blood pressure (DBP) (W = 491.500, p = 0.016). Lactate clearance was defined as the percent decrease in lactate from arrival in the ED to two hours after initiation of resuscitation. The lactate clearance percentage that was statistically significant is the difference between the survivors and non-survivors. The analysis showed a significant difference in lactate clearance (%) between the two groups, with a t-value of -8.666 and a p-value of <0.001. The survivors had a higher mean lactate clearance percentage (20.46%) compared to the non-survivors (10.86%). This difference was statistically significant, indicating that lactate clearance percentage was associated with mortality. These associations are summarized in Table 4 (W refers to the test statistic from a non-parametric statistical test, the Wilcoxon-Mann-Whitney U Test).

**Table 4:** Association of various parameters with mortality. SOFA: sequential organ failure assessment; qSOFA: quick sequential organ failure assessment; MEWS: modified early warning score; GCS: Glasgow coma scale; BPM: beats per minute; mm Hg: millimeters of mercury; PP: pulse pressure; MAP: mean arterial pressure; RR: respiratory rate; SpO_2_: peripheral oxygen saturation; mmol/L: millimoles per liter; IVC: inferior vena cava; VTI: velocity time integral; VET: volume expansion test; BP: blood pressure ^1^t-test; ^2^Fisher's exact test; ^3^chi-squared test; ^4^Wilcoxon-Mann-Whitney U test

Parameters	Association with mortality, p-value	Strength of association
Age (years)	0.372^1^	Point-biserial correlation = 0.08 (little/no association)
Gender	0.348^3^	Bias corrected Cramer's V = 0 (little/no association)
Comorbidities	0.162^2^	Bias corrected Cramer's V = 0.19 (low association)
SOFA	0.315^4^	Point-biserial correlation = 0.09 (little/no association)
qSOFA	<0.001^4^	Point-biserial correlation = 0.35 (medium effect size)
MEWS	<0.001^4^	Point-biserial correlation = 0.54 (large effect size)
GCS	<0.001^4^	Point-biserial correlation = 0.38 (large effect size)
Temperature (degree Celsius)	0.264^4^	Point-biserial correlation = 0.09 (little/no association)
Heart rate (BPM) (arrival)	0.080^4^	Point-biserial correlation = 0.18 (small effect size)
Systolic BP (mmHg) (arrival)	0.001^4^	Point-biserial correlation = 0.29 (medium effect size)
Diastolic BP (mmHg) (arrival)	0.016^4^	Point-biserial correlation = 0.28 (medium effect size)
PP (mmHg) (arrival)	0.052^4^	Point-biserial correlation = 0.2 (small effect size)
MAP (mmHg) (arrival)	0.001^4^	Point-biserial correlation = 0.3 (medium effect size)
RR (per minute) (arrival)	0.001^4^	Point-biserial correlation = 0.35 (medium effect size)
SpO_2_ (%) (arrival)	0.098^4^	Point-biserial correlation = 0.15 (small effect size)
Lactate (mmol/L) (arrival)	0.875^1^	Point-biserial correlation = 0.02 (little/no association)
IVC diameter (expiration) (cm) (arrival)	0.695^1^	Point-biserial correlation = 0.03 (little/no association)
IVC diameter (inspiration) (cm) (arrival)	0.071^4^	Point-biserial correlation = 0.16 (small effect size)
Caval index (%) (arrival)	0.128^4^	Point-biserial correlation = 0.17 (small effect size)
VTI (cm) (arrival)	0.773^4^	Bias corrected Cramer's V = 0.14 (low association)
Heart rate (BPM) (after VET)	0.646^1^	Point-biserial correlation = 0.08 (little/no association)
Systolic BP (mmHg) (after VET)	0.226^4^	Point-biserial correlation = 0.17 (small effect size)
Diastolic BP (mmHg) (after VET)	0.306^4^	Point-biserial correlation = 0.02 (little/no association)
PP (mmHg) (after VET)	0.427^4^	Point-biserial correlation = 0.04 (little/no association)
MAP (mmHg) (after VET)	0.245^4^	Point-biserial correlation = 0.03 (little/no association)
RR (per minute) (after VET)	0.001^4^	Point-biserial correlation = 0.34 (medium effect size)
SpO_2_ (%) (after VET)	0.160^4^	Point-biserial correlation = 0.14 (small effect size)
Lactate (mmol/L) (after VET)	0.025^1^	Point-biserial correlation = 0.21 (small effect size)
Lactate clearance (%)	<0.001^1^	Point-biserial correlation = 0.41 (large effect size)
IVC diameter (expiration) (cm) (after VET)	0.944^4^	Point-biserial correlation = 0.03 (little/no association)
IVC diameter (inspiration) (cm) (after VET)	0.713^4^	Point-biserial correlation = 0.08 (little/no association)
Caval index (%) (after VET)	0.765^1^	Point-biserial correlation = 0.03 (little/no association)
VTI (cm) (after VET)	0.934^4^	Point-biserial correlation = 0.02 (little/no association)
Source of sepsis	0.601^3^	Point-biserial correlation = 0.02 (little/no association)
Change in VTI (%)	0.489^4^	Point-biserial correlation = 0.02 (little/no association)
Fluid response status	0.617^3^	Point-biserial correlation = 0.02 (little/no association)

Among the 113 participants, the VET classified 50 (44.2%) as responders and 63 (55.8%) as non-responders based on their caval index. No significant differences were observed in baseline laboratory parameters, sources of sepsis, or outcomes between the two groups. The details are summarized in Table 5.

**Table 5:** Comparison of the baseline variables, basic lab parameters, source of sepsis and outcomes between responders and non-responders SOFA: sequential organ failure assessment; qSOFA: quick sequential organ failure assessment; MEWS: modified early warning score; mmol/L: millimoles per liter; VET: volume expansion test; g/dL: gram per deciliter; mg/dL: milligrams per deciliter; ng/mL: nanograms per milliliter; UTI: urinary tract infection; NSTI: necrotizing soft tissue infection; ICU: intensive care unit ^1^t-test; ^2^Fisher's exact test; ^3^chi-squared test; ^4^Wilcoxon-Mann-Whitney U test

Parameters	Fluid response status		p-value
	Responder (n = 50)	Non-responder (n = 63)	
Age	48.62 ± 17.62	48.75 ± 16.29	0.969^1^
Age			
18-30 years	11 (22.0%)	11 (17.5%)	
31-40 years	8 (16.0%)	11 (17.5%)	
41-50 years	5 (10.0%)	12 (19.0%)	
51-60 years	10 (20.0%)	10 (15.9%)	
61-70 years	10 (20.0%)	14 (22.2%)	
71-80 years	6 (12.0%)	3 (4.8%)	
81-90 years	0 (0.0%)	2 (3.2%)	
Gender			0.905^3^
Male	32 (64.0%)	41 (65.1%)	
Female	18 (36.0%)	22 (34.9%)	
Comorbidities			0.245^2^
None	17 (34.0%)	31 (49.2%)	
Hypertension	12 (24.0%)	5 (7.9%)	
Diabetes mellitus, hypertension	7 (14.0%)	7 (11.1%)	
Diabetes mellitus	6 (12.0%)	7 (11.1%)	
Coronary artery disease	4 (8.0%)	4 (6.3%)	
Hypothyroidism	3 (6.0%)	5 (7.9%)	
Chronic liver disease	1 (2.0%)	4 (6.3%)	
SOFA	10.82 ± 1.55	10.79 ± 1.52	0.899^4^
qSOFA	1.80 ± 0.99	1.76 ± 0.98	0.840^4^
MEWS	6.78 ± 1.81	6.71 ± 1.66	0.631^4^
Lactate (mmol/L) (arrival)	4.14 ± 0.61	4.08 ± 0.64	0.648^1^
Lactate (mmol/L) (after VET)	3.37 ± 0.71	3.32 ± 0.67	0.680^1^
Lactate clearance (%)	19.10 ± 8.70	19.09 ± 7.72	0.831^4^
Hemoglobin (g/dl)	11.61 ± 2.45	11.25 ± 2.19	0.399^4^
Platelet (10³/mm³)	115.38 ± 52.60	108.27 ± 56.40	0.472^4^
TLC (10³/mm³)	17.06 ± 5.56	16.54 ± 6.08	0.864^4^
Urea (mg/dl)	91.72 ± 29.32	92.78 ± 34.87	0.952^4^
Creatinine (mg/dL)	3.02 ± 1.31	2.85 ± 1.23	0.560^4^
Total Bilirubin (mg/dL)	2.90 ± 1.28	3.21 ± 1.21	0.211^4^
PCT (ng/ml)	27.44 ± 10.80	26.08 ± 11.05	0.510^1^
Source of sepsis			0.115^2^
Pneumonia	18 (36.0%)	32 (50.8%)	
UTI	10 (20.0%)	13 (20.6%)	
Neutropenic infection	4 (8.0%)	8 (12.7%)	
Bloodstream infection	6 (12.0%)	5 (7.9%)	
Intra-abdominal infection	7 (14.0%)	1 (1.6%)	
Meningitis	2 (4.0%)	3 (4.8%)	
NSTI	3 (6.0%)	1 (1.6%)	
Outcome			0.428^3^
Discharge	14 (28.0%)	16 (25.4%)	
ICU admission	15 (30.0%)	18 (28.6%)	
Need for mechanical ventilation	6 (12.0%)	16 (25.4%)	
Need for hemodialysis	7 (14.0%)	5 (7.9%)	
Dead	8 (16.0%)	8 (12.7%)	
Mortality	8 (16.0%)	8 (12.7%)	0.617^3^

After the VET, significant differences in vital signs emerged: responders had higher median SBP (W = 2998.000, p < 0.001; point-biserial correlation = 0.74), DBP (W = 3082.500, p < 0.001; correlation = 0.89), and MAP (W = 3098.000, p < 0.001; correlation = 0.88), indicating large effect sizes.

We observed some significant differences in POCUS parameters following VET. A smaller median IVC diameter was observed during inspiration in the responders (W = 32.000, p < 0.001; correlation = 0.87). The median (IQR) IVC diameter during inspiration was 0.9 (0.8-1.1) cm in responders and 1.4 (1.3-1.4) cm in non-responders. Additionally, responders demonstrated a higher median VTI (W = 2340.000, p < 0.001; correlation = 0.21). The median (IQR) VTI value was 17.65 (16.2-18.75) cm in responders and 16.1 (14.85-16.8) cm in non-responders. The median caval index was also higher in responders (W = 3150.000, p < 0.001; point-biserial correlation = 0.82-large effect size). The median (IQR) caval index after VET was 52.3 (43.12-61.12) percentage in responders and 26.7 (18.8-35) percentage in non-responders. Furthermore, responders showed a greater median change in VTI (W = 3126.000, p < 0.001; correlation = 0.93). The median (IQR) change in VTI (%) was 16.35 (15.72-17.71) percentage in responders and 5 (4.79-5.35) percentage in non-responders. Table 6 provides a summary of the comparison of vital parameters and POCUS findings at arrival and after VET between responders and non-responders.

**Table 6:** Comparison of vitals parameters and POCUS findings at arrival and after volume expansion test between responders and non-responders BPM: beats per minute; BP: blood pressure; mm Hg: millimeters of mercury; PP: pulse pressure; MAP: mean arterial pressure; RR: respiratory rate; SpO_2_: peripheral oxygen saturation; GCS: Glasgow coma scale; PaO_2_: partial pressure of oxygen in arterial blood; FiO_2_: fractional inspired oxygen; IVC: inferior vena cava; VTI: velocity time integral; LVOT: left ventricular outflow tract; mL: milliliters; VET: volume expansion test ^*^significant at p<0.05, ^1^t-test; ^2^Fisher's exact test; ^3^chi-squared test; ^4^Wilcoxon-Mann-Whitney U test

Parameters	Fluid response status		p-value	Strength of correlation (point-biserial correlation)
	Responder (n = 50)	Non-Responder (n = 63)		
Heart rate (BPM) (arrival)	122.24 ± 12.38	122.21 ± 11.48	0.988^1^	0 (little/no association)
Systolic BP (mmHg) (arrival)	73.20 ± 8.83	72.32 ± 9.18	0.605^1^	0.05 (little/no association)
Diastolic BP (mmHg) (arrival)	41.32 ± 4.40	40.98 ± 5.04	0.597^4^	0.04 (little/no association)
PP (mmHg) (arrival)	31.88 ± 5.63	31.33 ± 6.78	0.818^4^	0.04 (little/no association)
MAP (mmHg) (arrival)	51.95 ± 5.65	51.43 ± 5.91	0.580^4^	0.04 (little/no association)
RR (per minute) (arrival)	22.54 ± 4.16	23.32 ± 4.41	0.359^4^	0.09 (little/no association)
SpO_2_ (%) (arrival)	78.76 ± 12.64	75.65 ± 14.82	0.299^4^	0.11 (small effect size)
GCS	13.78 ± 1.17	14.02 ± 0.92	0.383^4^	0.11 (small effect size)
Temperature (degree Celsius)	37.11 ± 1.19	37.10 ± 1.12	0.944^4^	0 (little/no association)
PaO_2_/FiO_2_ (%)	358.30 (54.97)	349.51 (61.33)	0.603	0.07 (little/no association)
IVC diameter (expiration) (cm) (arrival)	1.61 ± 0.24	1.64 ± 0.27	0.604^1^	0.05 (little/no association)
IVC diameter (inspiration) (cm) (arrival)	0.69 ± 0.23	0.64 ± 0.20	0.296^4^	0.13 (small effect size)
Caval index (%) (arrival)	56.24 ± 13.90	60.01 ± 13.23	0.119^4^	0.14 (small effect size)
VTi (cm) (arrival)	15.49 ± 2.52	15.89 ± 3.04	0.534^4^	0.07 (little/no association)
LVOT diameter (cm)	1.88 ± 0.17	1.82 ± 0.15	0.053^4^	0.18 (small effect size)
Stroke volume (mL)	43.12 ± 9.05	41.77 ± 10.72	0.167^4^	0.07 (little/no association)
Cardiac output (mL/minute)	5266.91 ± 1198.48	5098.71 ± 1346.81	0.275^4^	0.07 (little/no association)
Heart rate (BPM) (after VET)	105.28 ± 10.15	108.97 ± 10.69	0.064^1^	0.17 (small effect size)
Systolic BP (mmHg) (after VET)*	89.84 ± 5.51	72.57 ± 9.29	<0.001^4^	0.74 (large effect size)
Diastolic BP (mmHg) (after VET)*	76.16 ± 6.24	51.37 ± 6.24	<0.001^4^	0.89 (large effect size)
PP (mmHg) (after VET)*	13.68 ± 3.17	21.21 ± 7.89	<0.001^4^	0.52 (large effect size)
MAP (mmHg) (after VET)*	80.72 ± 5.82	58.43 ± 6.40	<0.001^4^	0.88 (large effect size)
RR (per minute) (after VET)	17.98 ± 4.08	18.94 ± 4.54	0.285^4^	0.11 (small effect size)
SpO_2 _(%) (after VET)	84.84 ± 11.89	81.87 ± 13.74	0.224^4^	0.11 (small effect size)
IVC diameter (expiration) (cm) (after VET)	1.97 ± 0.32	1.91 ± 0.27	0.200^4^	0.1 (small effect size)
IVC diameter (inspiration) (cm) (after VET)*	0.93 ± 0.17	1.38 ± 0.09	<0.001^4^	0.87 (large effect size)
Caval index (%) (after VET)*	52.28 ± 9.38	26.62 ± 8.61	<0.001^4^	0.82 (large effect size)
VTi (cm) (after VET)*	18.04 ± 2.92	16.69 ± 3.20	<0.001^4^	0.21 (small effect size)

Diagnostic performance of LVOT VTI in predicting fluid response status

**Figure 3:**
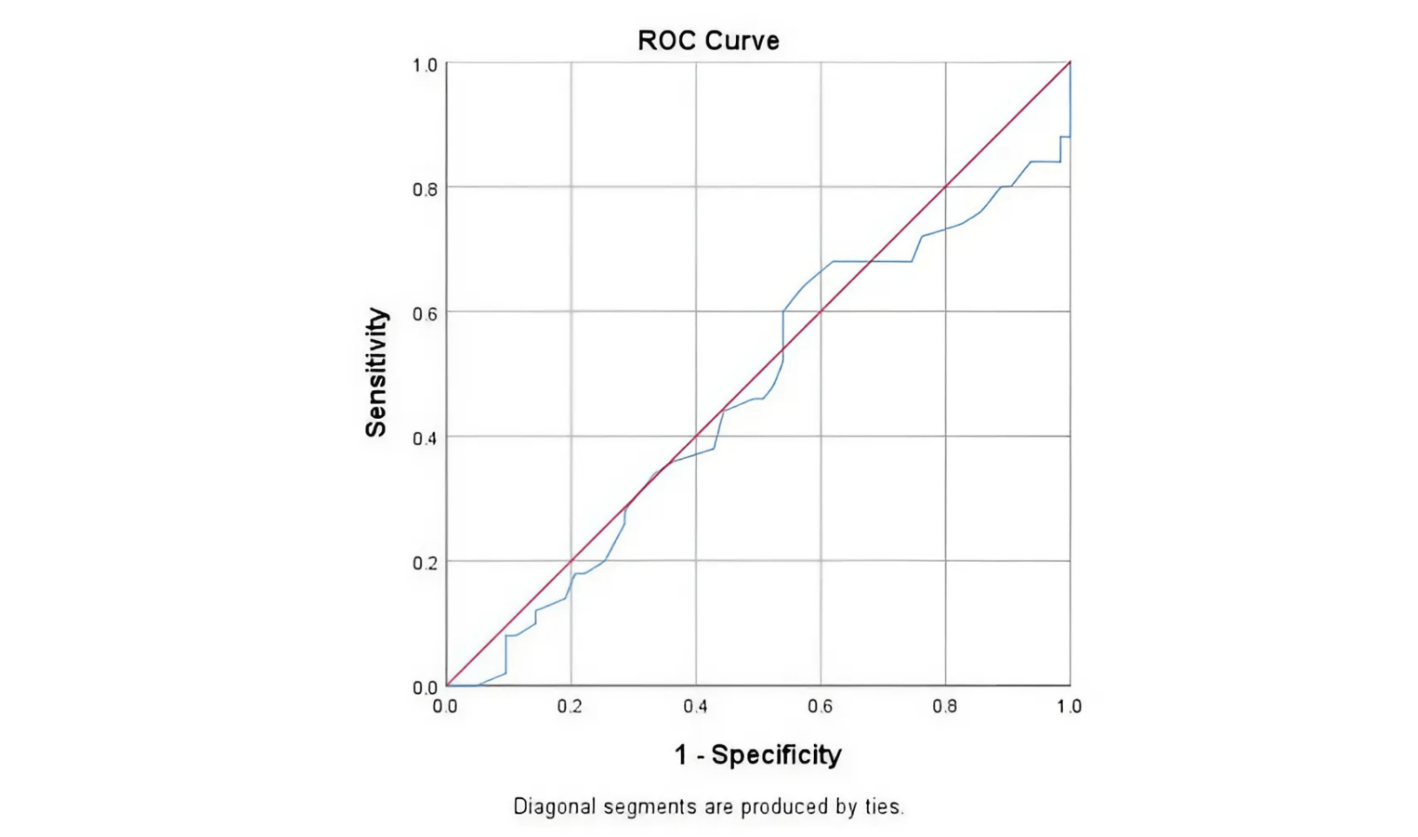
ROC curve analysis showing diagnostic performance of LVOT VTI (cm) on arrival in predicting fluid responsiveness ROC: receiver operating characteristic

The AUROC for LVOT VTI measured at arrival for predicting fluid responsiveness was 0.466 (95% CI: 0.372-0.576), indicating poor diagnostic capability (p = 0.466). (Figure 3 shows the ROC curve analysis of the diagnostic performance of LVOT VTI (cm) on arrival in predicting fluid responsiveness, n = 113). Using a cutoff of LVOT VTI ≤13.05 cm, sensitivity was 94% (95% CI: 83-98) and specificity was 100% (95% CI: 91-100) for predicting a caval index ≥40% after volume expansion testing (VET) (Table 7).

**Table 7:** Details of the ROC of LVOT VTI (at arrival) and change in LVOT VTI (%) LVOT: left ventricular outflow tract; VTI: velocity time integral; ROC: receiver operating characteristic; AUROC: area under the receiver operating characteristic

Parameter	LVOT VTI value (95% CI)	Change In LVOT VTI (%) value (95% CI)
Cutoff (p-value)	≤13. 05 (0.466)	≥15.19 (<0.001)
AUROC	0.466 (0.372-0.576)	0.992 (0.979-1)
Sensitivity	94.0% (83-98)	96.0% (86-100)
Specificity	100.0% (91-100)	100.0% (94-100)
Diagnostic accuracy	61.9%	98.2% (94-100)

**Figure 4:**
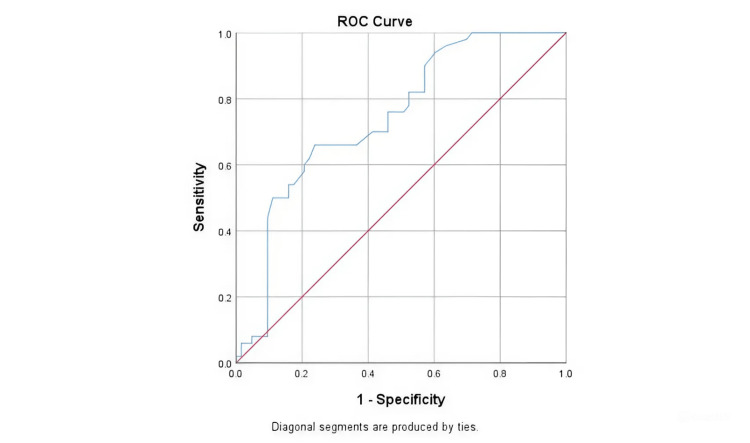
ROC curve analysis showing diagnostic performance of LVOT VTI (cm) after VET in predicting fluid responsiveness ROC: receiver operating characteristic; LVOT: left ventricular outflow tract; VTI: velocity time integral; VET: volume expansion test

Figure 4 shows the ROC curve analysis of the diagnostic performance of LVOT VTI (cm) after VET in predicting fluid responsiveness (n = 113). The AUROC for LVOT VTI measured after VET for predicting fluid responsiveness was 0.743 (95% CI: 0.652-0.834).

**Figure 5:**
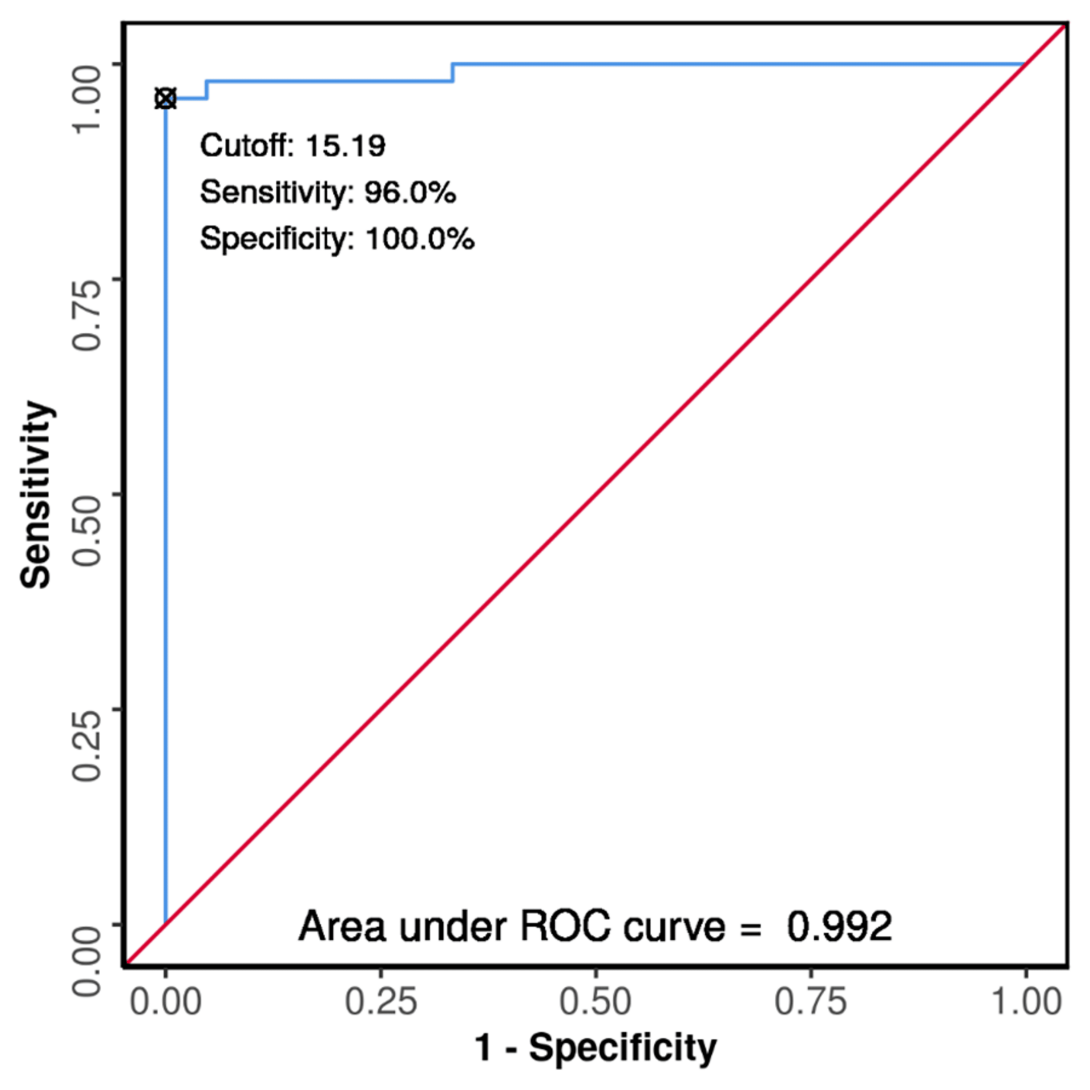
ROC curve analysis showing diagnostic performance of Change In LVOT VTI (%) in predicting fluid responsiveness LVOT: left ventricular outflow tract; VTI: velocity time integral; ROC: receiver operating characteristic

In contrast, the AUROC for the percentage change in LVOT VTI for predicting fluid responsiveness was 0.992 (95% CI: 0.979-1), demonstrating excellent diagnostic performance and statistical significance (p < 0.001). (Figure 5 shows the ROC curve analysis of the diagnostic performance of the change in LVOT VTI (%) in predicting fluid responsiveness, n = 113). At a cutoff of a change in LVOT VTI (%) ≥ 15.19, sensitivity was 96% and specificity was 100% for predicting fluid responsiveness (Table 7).

The following variables were significantly associated (p < 0.05) with change in VTI (%) - SBP after VET (mmHg), diastolic after VET (mmHg), PP after VET (mmHg), MAP after VET (mmHg), IVC diameter in inspiration after VET (cm), and caval index (%) after VET. Table 8 provides a summary of these associations.

**Table 8:** Association of various parameters with change in VTI (%) SOFA: sequential organ failure assessment; qSOFA: quick sequential organ failure assessment; MEWS: modified early warning score; BPM: beats per minute; BP: blood pressure; mm Hg: millimeters of mercury; PP: pulse pressure; MAP: mean arterial pressure; RR: respiratory rate; SpO_2_: peripheral oxygen saturation; GCS: Glasgow coma scale; IVC: inferior vena cava; VTI: velocity time integral; LVOT: left ventricular outflow tract; mL: milliliters; VET: volume expansion test *significant at p<0.05, ^1^t-test; ^2^Fisher's exact test; ^3^chi-squared test; ^4^Wilcoxon-Mann-Whitney U test

Parameters	Correlation coefficient (rho)	p-value
Age (years)	0.04	0.688^1^
SOFA	0.03	0.749^1^
qSOFA	0.13	0.177^1^
MEWS	0.13	0.175^1^
GCS	-0.1	0.315^1^
Temperature (degree Celsius)	-0.04	0.696^1^
Heart rate (BPM) (arrival)	0.01	0.902^1^
Systolic BP (mmHg) (arrival)	-0.04	0.700^1^
Diastolic BP (mmHg) (arrival)	-0.06	0.554^1^
PP (mmHg) (arrival)	0.03	0.792^1^
MAP (mmHg) (arrival)	-0.06	0.547^1^
RR (per minute) (arrival)	0.05	0.626^1^
SpO_2_ (%) (arrival)	0.05	0.590^1^
Lactate (mmol/L) (arrival)	0.01	0.944^1^
IVC diameter (expiration) (cm) (arrival)	-0.07	0.471^1^
IVC diameter (inspiration) (cm) (arrival)	0.09	0.330^1^
Caval index (%) (arrival)	-0.16	0.089^1^
Heart rate (BPM) (after VET)	-0.14	0.129^1^
Systolic BP (mmHg) (after VET)*	0.67	<0.001^1^
Diastolic BP (mmHg) (after VET)*	0.73	<0.001^1^
PP (mmHg) (after VET)*	-0.58	<0.001^1^
MAP (mmHg) (after VET)*	0.72	<0.001^1^
RR (per minute) (after VET)	0.01	0.899^1^
SpO_2_ (%) (after VET)	0.08	0.424^1^
Lactate (mmol/L) (after VET)	0	0.976^1^
Lactate clearance (%)	0.05	0.620^1^
IVC diameter (expiration) (cm) (after VET)	0.09	0.342^1^
IVC diameter (inspiration) (cm) (after VET)*	-0.75	<0.001^1^
Caval index (%) (after VET)*	0.76	<0.001^1^

## Discussion

In our observational study, we included 113 patients with a mean age of 48.69 years (SD: 16.81), predominantly male (64.6%). In comparison, Todi et al. reported an average age of 58.17 years with 57.71% male participants [13], while Chatterjee et al. found a mean age of 59.4 years and 56.8% male subjects [14]. In our study, 42.5% of patients had no comorbidities, with hypertension being the most prevalent at 15.0%, followed by diabetes at 12.4%, and both conditions combined at 11.5%. A Spanish study also reported high rates of hypertension and diabetes [15], and Verma and Kumar identified diabetes (24.8%) and COPD (13.1%) as significant comorbidities in septic shock patients [16]. Our cohort had a mean SOFA score of 10.81 (±1.52) and a mean qSOFA score of 1.78 (±0.98), which were lower compared to the SOFA score (26.18 ± 15.83) reported by Raibhoge and Mali [17] and the mean qSOFA score of 2.16 (±0.37) found by Nagdev et al. [18]. These lower scores suggest our patients were in the earlier stages of shock. Among our participants, 29.2% required ICU admission, 19.5% needed mechanical ventilation, and 10.6% required hemodialysis. The overall mortality rate was 14.2%, lower than the 34.7% by Bauer et al. [2], 50.8% reported by Todi et al. [19], 57.6% by Todi et al. [13], and 62.8% by Chatterjee et al. [14], likely due to our inclusion of patients in the early phases of shock. Lung infections were the primary cause of sepsis in our study, affecting 50 patients (44.2%), followed by UTIs in 23 patients (20.4%) and neutropenic infections in 12 patients (10.6%).

Our analysis found significant correlations between qSOFA, MEWS scores, and mortality. Majmundar et al. showed MEWS ≥5 was a better predictor of ICU mortality than NEWS ≥5, qSOFA ≥2, and SIRS ≥2 [20]. Sanguanwit et al. reported that qSOFA ≥2 outperformed other scores in predicting 28-day mortality and septic shock in elderly patients [21]. Lactate clearance differed significantly between mortality groups, with survivors showing higher rates. Filho et al. identified lactate >2.5 mmol/L as a strong predictor of 28-day mortality in severe sepsis [22], while Lee et al. found six-hour lactate levels better predicted 30-day mortality than initial levels or clearance [23]. Our study, however, did not find a significant link between initial lactate levels and mortality.

No significant differences were observed between responders and non-responders in terms of age, gender, comorbidities, or baseline SOFA, qSOFA, and MEWS scores. Additionally, baseline vital signs - HR, SBP, DBP, MAP, and initial POCUS parameters - were similar across both groups. After volume expansion, significant differences were observed between the groups in SBP, DBP, and MAP (p < 0.005). POCUS parameters, including IVC diameter during inspiration, caval index (%), VTI (cm), and the percentage change in VTI, also showed statistically significant differences. These findings underscore the utility of these measurements in distinguishing fluid responders from non-responders.

The normal range for LVOT VTI is typically between 18 cm and 22 cm, and for HRs, it is between 55 bpm and 95 bpm [10]. In shock, VTI usually drops below 15 cm. In our study, the mean VTI at arrival was 15.71 ± 2.82 cm, with some participants exceeding 22 cm, suggesting early-stage shock. The percentage change in LVOT VTI (%) which was calculated as the difference between VTI values before and after volume expansion divided by the VTI on arrival multiplied by 100, demonstrated excellent diagnostic performance, with a sensitivity of 96.0%, specificity of 100%, and an area under the ROC curve (AUC) of 0.992. The optimal cutoff for the percentage change in VTI was ≥15.19%, aligning with findings from Blanco et al. [10] and Wang et al. [24], who emphasized the strong predictive value of delta VTI. A single VTI measurement was less effective, with an area under the ROC of 0.466, which highlights that the changes in VTI are more relevant for evaluating fluid responsiveness. Variations in LVOT VTI directly influence stroke volume, reinforcing its role as a surrogate marker for stroke volume [10].

While the predictive value of LVOT VTI for fluid responsiveness is well-established, much of the research has focused on ICU or operating theatre settings [7,8,25,26]. Most studies emphasize the use of delta VTI as a predictor of fluid responsiveness, calculated using the formula:\[ \Delta \text{VTI} (\%) = \frac{\text{VTI}_{\text{max}} - \text{VTI}_{\text{min}}}{\frac{\text{VTI}_{\text{max}} + \text{VTI}_{\text{min}}}{2}} \times 100\% \] where VTI_max_ and VTI_min_ are the maximum and minimum VTI measured over 10 cardiac cycles [7,24,27]. However, measuring delta VTI can be time-consuming, requiring multiple POCUS measurements over 10 cardiac cycles, which complicates its use in emergency settings.

Our study has several limitations. Firstly, we excluded mechanically ventilated patients, which limits the generalizability of our findings to this critical patient population. Additionally, the absence of a universally accepted gold standard for assessing fluid responsiveness complicates the validation of our LVOT VTI measurements. The relatively small sample size also restricts the robustness of our results, highlighting the need for larger, multicentric studies to confirm our findings. Observer bias was another concern, as the lack of blinding may have impacted the objectivity of the results. Finally, given our focus on patients with early-stage shock, our findings may not be applicable to those with undifferentiated or severe shock.

## Conclusions

In evaluating patients with sepsis-related acute circulatory failure in the emergency department, LVOT VTI plays a key role in assessing fluid responsiveness. Our study demonstrated that the percentage change in LVOT VTI after a VET exhibited excellent diagnostic performance, with a sensitivity of 96.0%, specificity of 100%, and an area under the ROC curve of 0.992. A change of ≥15.19% indicates a high probability of fluid responsiveness. In contrast, a single VTI measurement was less reliable, with poor diagnostic capability. We also observed significant associations between the percentage change in VTI and factors like MAP, IVC diameter, and caval index post-volume expansion, all correlating with fluid responsiveness.

Unlike delta VTI, which requires time-consuming measurements over 10 cardiac cycles, the percentage change in VTI offers a more efficient means of evaluation. Moreover, our analysis revealed strong correlations between qSOFA and MEWS scores with mortality, with lactate clearance notably higher in survivors.
